# The social cognitive and neural mechanisms that underlie social functioning in individuals with schizophrenia – a review

**DOI:** 10.1038/s41398-023-02593-1

**Published:** 2023-10-21

**Authors:** Imke Lemmers-Jansen, Eva Velthorst, Anne-Kathrin Fett

**Affiliations:** 1 Department of Clinical, Neuro and Developmental Psychology, Faculty of Behavioural and Movement Sciences, Institute for Brain and Behaviour (iBBA) Amsterdam, Vrije Universiteit Amsterdam, Amsterdam, The Netherlands; 2 Department of Psychosis Studies, Institute of Psychiatry, Psychology and Neuroscience, King’s College London, London, UK; 3 GGZ Noord-Holland-Noord, Heerhugowaard, The Netherlands; 4 Department of Psychology, City, University of London, London, UK

**Keywords:** Schizophrenia, Human behaviour

## Abstract

In many individuals with a diagnosis of schizophrenia social functioning is impaired across the lifespan. Social cognition has emerged as one of the possible factors that may contribute to these challenges. Neuroimaging research can give further insights into the underlying mechanisms of social (cognitive) difficulties. This review summarises the evidence on the associations between social cognition in the domains of theory of mind and emotion perception and processing, and individuals’ social functioning and social skills, as well as associated neural mechanisms. Eighteen behavioural studies were conducted since the last major review and meta-analysis in the field (inclusion between 7/2017 and 1/2022). No major review has investigated the link between the neural mechanisms of social cognition and their association with social functioning in schizophrenia. Fourteen relevant studies were included (from 1/2000 to 1/2022). The findings of the behavioural studies showed that associations with social outcomes were slightly stronger for theory of mind than for emotion perception and processing. Moreover, performance in both social cognitive domains was more strongly associated with performance on social skill measures than questionnaire-based assessment of social functioning in the community. Studies on the underlying neural substrate of these associations presented mixed findings. In general, higher activation in various regions of the social brain was associated with better social functioning. The available evidence suggests some shared regions that might underlie the social cognition-social outcome link between different domains. However, due to the heterogeneity in approaches and findings, the current knowledge base will need to be expanded before firm conclusions can be drawn.

## Introduction

Impaired social functioning is a chronic and relatively stable feature of schizophrenia and related disorders (SZ) [1, 2]. Remarkably, despite the undeniable importance of close social connections for physical [3] and mental [4] health, social functioning remains among the least studied characteristics of SZ, resulting in an important gap of knowledge concerning its underlying mechanisms. A key goal in the field is therefore to identify treatable determinants of poor social outcomes.

In the past two decades, impairments in social cognition (SC), referring to the psychological processes that enable people to understand other’s social behaviour, have emerged as some of the possible factors that may underlie difficulties in social functioning [5–7]. SC is often impaired in SZ, with reported effect sizes ranging from *d* = 0.88 to 1.04 for different SC domains [8]. Importantly, while effect sizes of impaired neurocognitive functioning seem to exceed those for SC functioning [9], two large systematic reviews and meta-analyses suggest that SC may explain more unique variance in functional outcome [5, 6]. In general, SC impairments have been found to be largely independent of clinical symptoms, present before the onset of illness and relatively stable over time, and as such fulfil the criteria of a potential treatment target [5, 6, 10, 11]. However, to determine the full potential of SC as target for interventions in SZ, several issues need to be addressed.

First, SC is a multi-dimensional construct that has been conceptualised into different cognitive functions such as: (1) theory of mind (ToM), also referred to as mentalising, cognitive empathy, or mental state attribution, including the ability to take others’ perspectives, to represent their mental states, intentions, beliefs, or dispositions; (2) emotional perception and processing (EPP), i.e., perception, use, and understanding of emotional information, including the recognition of different emotions from facial expressions, body posture or voices; (3) social perception and knowledge (SP), i.e., the ability to decode and interpret social cues, to process social context and social knowledge of rules, roles, and goals; and (4) attributional bias (AB), i.e., the reasoning about causes of social events or interactions [12, 13]. Crucially, not every domain may impact social outcomes equally, and it is still unclear which domain may be most relevant to social outcomes [14]. Earlier work showed associations between SC and a variety of broader community outcomes that were in the upper small to large range, with the largest effect size correlations for ToM (*ûp* = 0.48), followed by SP, and EPP [5]. ToM has therefore been suggested to be particularly important when it comes to functioning in the community, although newer evidence shows more modest associations (*ûp* = 0.21) [6].

Second, ‘social outcome’ is a broad construct, often used interchangeably for distal outcomes, such as community functioning (including interpersonal functioning, functioning at school, work, activities, self-care, and various independent living skills) and more proximal outcomes, such as social behaviour in the milieu, social problem-solving skills, and social skills, which are typically assessed through observation or in controlled lab settings. It is unclear which (if any) social outcome may predominantly benefit from the improvement of SC skills. Previous work suggests that SC processes, such as ToM, may be particularly important for community functioning and social skills. However, it remains unclear how ToM and other SC functions differentially relate to different social outcomes, such as real-life social functioning, social behaviour in the milieu, or indices of social capacity, including social problem-solving skills, or social skills (e.g., making eye contact, voice volume and tone).

Third, investigations of the underlying neural mechanisms of the relationships between SC and social functioning point to a wide variety of potentially relevant brain areas [15]. A widespread medial-frontoparietal ‘social brain’ network has been proposed to underlie various SC functions, consisting among others of the medial prefrontal cortex (mPFC), the temporo-parietal junction (TPJ), the posterior superior temporal sulcus (pSTS), the inferior frontal gyrus (IFG), the interparietal sulcus (IPS), the amygdala, the anterior (ACC) and posterior cingulate cortex (PCC) and the anterior insula (AI) [16–19]. In SZ, structural changes and aberrant activation of these brain regions have been reported in relation to SC task performance [15, 20, 21]. Most previous work focused on the neural mechanisms underlying ToM [22–27] and EPP [22, 27–31] and generally showed patterns of reduced brain activation in SZ, which, overall, appear to be associated with worse functional outcome [21]. In more complex social interaction and decision-making paradigms that rely on the integration of multiple SC functions, reduced activation has been found in the mPFC, insula and TPJ, which have been viewed as regions of the ‘social brain’. Additionally, reduced activation in the dorso-lateral prefrontal cortex (dlPFC) and basal ganglia has been reported, which among others have been associated with cognitive control, emotional processing and reward learning [32–37]. Some authors propose that more basic functions such as processing of salience, reward and embodiment may, at least partly, underlie SC deficits [38–41]. In SZ, smaller ventricles and greater grey matter volume in the cortex and fronto-limbic structures have been associated with better social functioning [15, 21]. However, insights into the neural mechanisms that underlie the associations between specific SC domains and different social outcomes are still scarce.

This systematic review and meta-analysis provide a state-of-the-art overview of the most recent studies on the relationships between SC and different social functional outcomes, as well as the underlying neural mechanisms of these associations.

## Method

The inclusion of articles on behavioural data was based on the search strategy of a recent meta-analysis by Halverson et al. [6], although here we focused on studies with predominantly social outcomes (vs. more general functioning in the community). Our searches were conducted in PubMed and spanned studies from July 2017 (i.e., studies not included in the meta-analysis of Halverson et al. [6]) to January 2022, using the search terms: *schizophrenia* combined with social functioning search terms *(social functioning, social skill, social behaviour, social adjustment, social dysfunction*) and social cognition search terms (emotion* perception, affect perception, affect recognition, emotion recognition, attribution*, theory of mind, mentalizing, mentalising, social cognition, prosody, social knowledge, mind reading, social cue, social judgement). To retrieve neuroimaging articles the search time frame was extended from January 2000 to January 2022 (given that a previous ALE meta-analysis identified the first SZ neuroimaging papers on ToM/EPP in 2002) [22], and combined the initial search with: *neuroimaging, fMRI, MRI. A second search for relevant neuroscientific literature was conducted using the search terms electroencephalogram (EEG), magnetoencephalography (MEG) and positron emission tomography (PET).

### Article inclusion criteria

Articles were included if they: were written in English language, included individuals with a diagnosis of non-affective psychosis, reported at least one cross-sectional correlation between one of the SC and social outcome domains specified below, and used established and reproducible SC and outcome measures that predominantly focused on different aspects of social outcomes.

#### Social cognition

For social cognition, we included the domains of theory of mind (ToM), emotion perception and processing (EPP), social perception and knowledge, attribution bias, and studies that reported a combined SC score based on these domains.

#### Social outcomes

We included studies that addressed *social functioning* (SF) through questionnaire assessment or observer ratings of relationship functioning (e.g., number of close friends, psychosexual relationships); *social behaviour in the milieu* through observed behaviours in a specific social context (e.g., social behaviour on the ward); *social problem solving*, (e.g., ability to generate solutions in social situations); and *social skills (*SS), (e.g., interaction abilities including conversation skills). Typically, social functioning and social behaviour in the milieu are based on ratings of real-world social behaviour, whereas social problem solving, and social skill are measures of social functional capacity that rely on task-based performance [6, 42].

#### Neuroimaging

We only included functional MRI (fMRI) studies that required the completion of a SC task during fMRI scanning. Imaging studies investigating various aspects of brain structure or resting state connectivity were excluded (for a review on the relationship between brain structure, resting state connectivity and functional outcomes see [15]). There were only few neuroimaging studies per SC-social outcome domain and results of the neuroimaging studies are therefore described in a narrative way.

### Screening process

The behavioural study search yielded 2537 hits, which were screened for articles suitable for inclusion by EV and AF. Of those, 119 were considered for inclusion after the initial screening. Consensus decisions were made on the inclusion of any inconsistently screened articles. At the second stage, 95 articles were excluded as they did not report cross-sectional SC-outcome associations or reported on SC or social functioning only. Further, two studies were not available [43, 44], and one study reported only significant associations and was therefore excluded [45]. We only retrieved a small number of articles each that assessed ‘overall’ SC (2) [46, 47], attribution bias (1), and social perception and knowledge (1) [48]. Therefore, these articles could not be included, and meta-analyses were only conducted for the domains ToM and EPP. Two studies had overlapping samples and were averaged for the analysis [49, 50]. The 18 included studies are shown in Table 1.

**Table 1:** Studies on social cognition and social functioning published between 2017 and 2022.

Authors (year)	*N*	Age (yrs)	Male	White	Education (yrs)	Illness Duration (yrs)	INP	Anti psychotics	SZ	SZAF	PSY NOS	Other	SC domain	SC Measure	Outcome domain	Measure	Correlation r between social outcome and SC task
		Mean (SD)	N, (%)	N, (%)	Mean (SD)	Mean (SD)	(%)	(%)	(%)	(%)	(%)	(%)					
Bechi et al. (2021) [92]	123	44.2 (11.31)	71 (57.7)	-	11.91(2.79)	20.29 (10.14)	-	-	100	0	0	0	ToM	Picture Sequencing Task	SF	Quality of Life Scale (QLS, interpersonal relations score)	0.19
Bonfils et al. (2019) [93]	49	22.4 (3.9)	34 (69)	15 (31)	12.8 (1.4)	-	0	-	100	0	0	0	EPP	•Prosody task •Facial Emotion Identification task (FEIT)	SF	•Global functioning scale (GFS) (social) •Role functioning scale (RFS) social (Immediate Social Network Relationships and Family Network Relationships)	GFS (social) Prosody *=* 0.17 FEI*T* = 0.21 RFS social Prosody = 0.38 FEIT = 0.28 RFS family: Prosody = 0.23 FEIT = 0.12
Brunet-Gouet et al. (2021) [94]	143	31.4 (8.2)	111 (77.6)	-	12.6 (2.4)	-	0	-	-	-	0	0	EPP ToM	EPP •Test de Reconnaissance des Émotions Faciales (TREF) ToM •V-Sir versailles-situational intention reading •V-Comics: Versailles Intention Attribution Task •SPEX-GA & BA: Social cognition Perception •Executive functions—Belief & Goal Attribution sensitivities	SF	PSP	TREF = 0.32 ToM – V-Sir = 0.29 V-comics1 = 0.38 V-comics2 = 0.25 Spex-ba1 = 0.15 Spex-ba2 = 0.08 Spex-ga1 = 0.19 Spex-ga2 = 0.03
Canty et al. (2021) [56]	26 (early SZ (ESZ)), 32 (chronic SZ (CSZ))	23.2 (2.02), 31.6 (9.0)	13 (50), 22 (68.8)	-	12.2 (2.02), 11.3 (1.7)	0.6 (1.21), 10.98 (6.16)	57.7, 46.9	-	100	0	0	0	ToM	•Empathy Quotient EQ •Virtual Assessment of mentalising Ability (VAMA) (reporting total score for both measures)	SF, SS	Social functioning Scale (SFS) Social skills performance assessment (SSPA)	EQ/VAMA - SFS: ESZ = 0.52 CSZ = 0.56 EQ/VAMA - SSPA: ESZ = 0.66 CSZ = 0.68
Corbera et al. (2021) [95]	46	29.7 (8.1)	31 (67.4)	25 (54.3)	-	-	-	-	100		0	0	ToM	Empathy Quotient EQ	SF	SFS	0.12
Deste et al. (2020) [49] includes data from SCOPE 3 and 5 Harvey et al. (2019) [50]	361	41.7	244 (67.6)	-	12.9 (2.3)	-	-	-	100		0	0	EPP ToM	EPP •Bell Lysaker Emotion Recognition Task (BLERT) •Penn Emotion Recognition Test (ER-40) ToM •Reading the mind in the Eyes test (RMET) •Awareness of Social Inferences Test (TASIT) •Hinting task (Hints)	SF	Specific level of functioning (SLOF, interpersonal relationships (IP))	EPP BLERT = 0.21 ER-40 = 0.12 ToM RMET = 0.10 TASIT = 0.15 Hints = 0.10
Engelstad et al. (2017) [96]	54	28.7 (8.3)	33 9 (61.1)	-	11.7 (2.6)	5.8 (6.0)	24.1	-	70.4	29.6	0	0	EPP	Emotional Biological Motion test	SS	Interpersonal problem solving skills (AIPSS)	0.27
Frajo-Apor et al. (2021) [97]	63	44.8 (10.1)	37 (58.7)	-	12.7 (3.1)	15.4 (10.4)	0	98.4	100	0	0	0	EPP	MSCEIT	SF	Berliner Lebensqualitäts-profil (BELP, social contact with friends and family)	Family = 0.36 Friends = 0.24 Days social contact outside treatment = 0.33
Gurcan et al. (2021) [98]	30	39.07 ± 9.48/42.43 ± 8.19	20 (71.4)	-	11.07 ± 3.05/10.64 ± 3.17	16.14 (8.42) 20.21(7.74)	0	-	-	-	-	-	ToM	•RMET •First order false belief •Second order false belief •Metaphor •Irony •Faux pas	SF	Social functioning assessment scales (SFAS)	RMET = 0.51 First orde*r* = 0.42 Second order = 0.28 Metaphor = 0.21 Irony = 0.22 Faux pas = 0.56
Hajduk et al. (2018) [99]	59	39.29 (9.79)	33 (56)	43 (73)	13.15 (2.74)	-	-	-	100	0	0	0	EPP	•Body emotion recognition (BR-100) •Emotion recognition task (ER-40)	SF	•SLOF (IP, social appropriateness) •PSP (personal and social relationships, social activities)	BR-100: SLOF IP = 0.02 SLOF SA = 0.16 PSP PSR = 0.08 PSP SA = 0.32 ER-40: SLOF IP = -0.12 SLOF SA = 0.11 PSP PSR = -0.02 PSP SA = 0.18
Hajduk et al. (2020) [100]	43	38.16 (9.45)	20 (60)	-	Elementary/high school no diploma 21%, High school with diploma or university 80%	11.33 (9.12)	-	-	72	28	0	0	EPP ToM	EPP •Emotion Recognition Test ToM •Hints	SF	PSP (social relationships and activities)	ER: Social relationships = 0.12 Social activities = 0.10 Hints: Social relationships = 0.41 Social activities = 0.41
Harvey et al. (2019) [50]	312	~42	~63.5	43-50%	12.47-13.07	-	-	~96	52	48	0	0	EPP ToM	EPP •BLERT •ER-40 ToM •RMET •TASIT •Hints	SF	SLOF (social acceptability (SA) and interpersonal functioning (IP))	BLERT SLOF IP = 0.29 SLOF SA = 0.14 ER-40 SLOF IP = 0.14 SLOF SA = 0.04 RMET: SLOF IP = 0.10 SLOF SA = 0.15 TASIT SLOF IP = 0.17 SLOF SA = 0.06 Hints SLOF IP = 0.21 SLOF SA = 0.01
Kolavrambath et al. (2020) [101]	27	31.07 (8.9)	14 (52)	0	Graduation 44.4%, Postgraduation 29.62%	7 (4.07)	mostly outpatient	-	-	-	-	-	EPP	Tool for Recognition of Emotions in Neuropsychiatric Disorders	SF	Groningen Social Disabilities Schedule	0.31
Kurtz et al. (2018) [102]	60	28.27 (6.88)	65%	-	4.43 (3.02)	3.41 (1.75)	0	98.3	90	10	0	0	EPP	•Penn Emotion Acuity Test (PEAT) •MSCEIT	SF	WHO-DAS (getting along)	PEAT = 0.15 MSCEIT = 0.14
Le et al. (2018) [103]	146	41.5 (9.6)	68%	56%	12.3 (1.7)	-	0	97	80	20	0	0	ToM	Hints	SS	Maryland Assessment of Social Competence (MACS)	0.41
Ludwig et al. (2017) [104]	38	23.5 (3.0)	33 (86.7)	29 (76.3)	14.03 (1.5)	<5	0	82.1	65.8	15.8	18.4	0	AB EPP ToM SP Trust	EPP •BLERT •ER-40 ToM •Hints •RMET •TASIT	SF, SS	SLOF SSPA	SLOF EP = -0.1, 0.1 ToM = 0.19, 0.4 EP = 0.02, = 0.20 SSPA EP = 0.27, 0.34 ToM = 0.34, 0.38, 0.48
Park & Choi (2018) [105]	39	40.8 (13.0)	30 (76.9)	38 (97.4)	12.2 (2.3)	-	0	-	59	41	0	0	EPP	Emotional Context Processing Scale	SS	MACS social competence	0.30
Sabharwal et al. (2021) [64]	47	45.3 (8.1)	63.8	39 (83.0)	-	-	-	48.9	40.4		59.6		EPP	The emotional face perception task [106]	SF	Social and Occupational Functioning Assessment scale (SOFAS) Social functioning, a composite of social activity, social initiative, and socio-sexual relations ratings of (QLS)	SOFAS = 0.46 QLS = 0.31

*AB* Attributional bias, *EPP* Emotion Perception and Processing, *INP* inpatient, *PSYNOS* Psychosis not otherwise specified, *SC* Social Cognition, *SF* Social Functioning, *SP* Social Perception, *SS* Social Skills, *SZ* Schizophrenia, *SZAF* Schizoaffective, *ToM* Theory of Mind.

The neuroimaging study search yielded 739 hits that were screened for articles suitable for inclusion by ILJ. Of those, 186 articles were examined for possible inclusion after the initial screening, inclusion was discussed with AF and EV. Examination of a review paper from 2017 [45] yielded one additional article which was not retrieved by the original search [51]. One further article did not measure ToM directly but rather the mirror neuron system, and we therefore excluded this study from our review [52]. The next selection resulted in the exclusion of 29 articles not involving fMRI analyses, 123 articles not reporting associations between brain activation and social outcome measures, and a further 21 studies that were reviews, meta-analyses, theoretical papers, or papers that did not investigate the included SC domains or SZ. The final selection of 14 included studies is shown in Table 2. The search with EEG, MEG and PET resulted in 185 hits, of which 5 seemed suitable for inclusion (screened by ILJ, inclusion discussed with AF). Two papers did not include social functional measures, two associated mismatch negativity (which is not social cognition) with social functioning measures, and one paper met all requirements [53]. However, social and non-social stimuli were not separated in the study, hence it was not included in the review.

**Table 2:** Neuroimaging studies on social cognition and social functioning published since 2000.

Authors (year)	N	Age (yrs)	Male	White	Education (yrs)	Illness duration (yrs)	INP	Anti psychotics	SZ	SZ AF	PSY NOS	SC domain	Measure - fMRI paradigm	SF domain	SF measure	Analysis	Associations brain activation with SF	Correlation (r) between social outcome and neural activation during SC task
		Mean (SD)	N (%)	N (%)	Mean (SD)	Mean (SD)	N, (%)	(%)	(%)	(%)	(%)							
Bartholomeusz et al. (2018) [57]	14	20.4 (3.4)	8 (57)	-	12.3 (1.5)	< 2 years	0	86	79	14	7	ToM	Attribution of intentions paradigm	SF	SOFAS	Whole brain	No significant relationships between hypoactivation of the right TPJ, right orbitofrontal cortex (OFC) and left prefrontal cortex (PFC)/ inferior frontal gyrus (IFG) with SOFAS.	n.r.
Bjorkquist et al. (2016) [61]	14	31.6 (7.5)	10 (71)	-	13.9 (3.0)	-	0	79	100	0	0	EPP	Emotional picture rating task	SF	QLS	ROI: bilateral amygdala, bilateral mPFC (medial frontal gyrus and ACC)	Weaker right amygdala-mPFC coupling during negative compared to neutral image perception associated with poorer social functioning.	Amygdala-mPFC coupling: 0.63, *p* = 0.021
Das et al. (2012) [51]	20	34.5 (8.4)	20 (100)	-	11.09 (1.77)	9.4 (6.5)	-	95	100	0	0	ToM	Triangles task	SF	Life Skills Profile (LSP)	Whole brain	Stronger activation of r IFG (and STG) associated with poorer LSP total score	r IFG - LSP = -0.53, *p* = 0.028 STG - LSP = -0.39, n.s.
Dodell-Feder et al. (2015) [59]	20 SZ/ SZ AF	38.8 (9.7)	12 (60)	-	15.0 (2.3)	17.1 (12.2)	-	-	80	20	0	ToM	ToM false belief task	SF	SAS, GFSS	ROI: r TPJ, l TPJ, dorso-medial prefrontal cortex (dmPFC), middlemPFC and ventro-medial prefrontal cortex (vmPFC). Additional whole brain	For all participants: Higher activity in mPFC and r TPJ associated with higher SF (SAS) and higher mPFC with higher SF (GFS). In patients: Higher activity in mPFC associated with better SF.	All participants: SAS mPFC = 0.56, *p* < 0.001 rTPJ = 0.33, *p* < 0.001 GFSS mPFC = 0.45, *p* < 0.001 In patients: mPFC SAS = 0.44, *p* < 0.05 GFSS =.11, n.s.
	18 HC	32.4 (12.1)	12 (66.7)		14.2 (2.6)													
Hanssen et al. (2022) [68]	23	39.9 (9.1)	19 (82.6)	-	-	-	-	96	73.9	17.4	8.7	Complex	Trust game	SF	Experience sampling method (ESM)	ROI: r caudate, r TPJ, mPFC, and l dlPFC. Additional whole brain	Lower perceived social exclusion was marginally significantly associated with higher caudate activation in the positive context. Higher perceived relationship quality was associated with higher mPFC activation.	Caudate and perceived social exclusion: *b* = −0.17, *p* = 0.07 mPFC and perceived relationship quality: *b* = 0.27, *p* < 0.05
Hyatt et al. (2020) [60]	30	26.0 (3.5)	19 (63)	-	-	-	-	-	100	0	0	ToM & AB	Domino task	SF	QLS	Independent component analysis, resulting in 10 default mode network (DMN) regions: PCC, precuneus, l/r TPJ, dmPFC, ACC, vmPFC, mPFC	No significant correlations with SF	n.r.
Lee et al. (2006) [58] ^a^	14	31.7 (7.3)	13 (93)	-	-	9.8 (5.4)	100	100	100	0	0	ToM	Empathic and forgivability judgements	SF	Life Skills Profile	Whole brain	Increased activation of the left mPFC associated at trend level with better SF	mPFC: 0.51, *p* = 0.06
Nelson et al. (2015) [62]	14	33.4 (9.3)	71.4	35.7%	13.5 (3.4)	-	-	~64	100	0	0	EPP	Emotional picture rating task	SF	QLS	ROI: bilateral ACC	Decreased ACC activation to pleasant images associated with poorer SF	n.r.
Pinkham et al. (2008) [69]	12 NP-SZ	28.0 (3.9)	100%	11 (92)	13.3 (2.1)	-	-	-	75 NP-SZ	25 NP-SZ AF	0	Complex	Trustworthi-ness/ Approachability Task	SF	Social Functioning Scale	ROI: bilateral amygdala, bilateral fusiform gyrus (FG), bilateral superior temporal sulcus (STS), bilateral vlPFC, and mPFC.	For all participants: Increased activation of bilateral FG, mPFC, bilateral vlPFC with better SF. Increased superior temporal sulcus (STS) activation with SF (non-surviving multiple testing). Paranoid group: Increased left amygdala activation with better SF, and in right FG at trend level.	All participants: FG left = 0.51, *p* = 0.001 right = 0.53, *p* < 0.001 mPFC = 0.42, *p* = 0.005 vlPFC left = 0.48, *p* = 0.002 right = 0.49, *p* = 0.001 STS left = 0.29, *p* = 0.045 right = 0.41, *p* = 0.007 Paranoid group: Amygdala = 0.50, *p* = 0.048 r FG = 0.44, *p* = 0.076
	12 P-SZ	26.4 (5.3)		10 (83)	13.3 (2.7)				66.7 P-SZ	33.3 P-SZ AF								
	12 HC	27.1 (4.0)		10 (83)	16.9 (2.0)													
Pinkham et al. (2011) [63]	35	36.5 (10.7)	17 (48)	16 (46)	13.2 (2.5)	15.2 (11.1)	-	91,4	88,6	11,4	0	EPP	Facial emotion identification	SF	Strauss-Carpenter Outcome Scale	ROI: bilateral amygdala	Increased left amygdala activation with better SF in the paranoid patient group.	Left amygdala*:* 0.58, *p* < 0.001
Sabharwal et al. (2021) [64]	47	45.3 (8.1)	30 (63.8)	39 (83)	-	-	-	48.9	40.4	0	59.6	EPP	Emotional face perception task	SF	SOFAS & QLS	ROI: fusiform face area (FFA), STS, occipital face area (OFA), amygdala, insula, IFG. Connectivity analysis, basis amygdala.	Negative correlation IFG, calcarine cortex (CC) and insula with QLS. Negative correlations left amygdala-IFG connectivity with SOFAS. SF with amygdala-insula connectivity.	QLS IFG = −0.53, *p* < 0.001 CC = −0.56, *p* = 0.001 Insula = −0.47, *p* = 0.001 Left amygdala-IFG connectivity with SOFAS = −0.41, *p* = 0.004 Amygdala-insula connectivity with SF = n.r.
Shin et al. (2015) [67]	17	31.0 (6.1)	11 (64.7)	-	13.8 (1.6)	10.9 (6.9)	0	100	100	0	0	EPP	Emotion perception task	SF	Strauss–Carpenter Level of Functioning Scale	Whole brain and ROI: dlPFC and STS	Left dlPFC activity in the inappropriate condition positively correlated with SF.	dlPFC with SF = 0.69, *p* = 0.05
Smith et al. (2015) [66]	30	33.6 (7.1)	18 (60)	13 (43)	-	13.6 (7.5)	0	-	100	0	0	EPP	Emotional perspective taking task	SF, SS	Social attainment (SLOF) & role play (social competence)	Whole brain. Correlations with anterior insula, IFG, superior motor area / anterior midcingulate cortex (SMA/aMCC), mPFC, right TPJ, and precuneus. Exploratory correlations	Right SMA/aMCC with SS and SF. Right precuneal sulcus with SF at trend level.	SMA/aMCC with SS = 0.46, *p* = 0.05 SMA/aMCC with SF = 0.46, *p* < 0.05 Precuneal sulcus with SF = 0.35, *p* = 0.07
Taylor et al. (2011) [65]	21	40.7 (9.3)	14 (67)	-	14.4 (2.6)	19.5 (12.3)	-	100	76	24	0	EPP	MSCEIT managing emotions Social appraisal task	SF	SAS	ROI: mPFC PPI with dACC	Poorer SF with higher activation in precuneus, lingual gyrus.	n.r.

^a^Studies used a 1.5 Tesla scanner. All other studies used 3 T scanners.

*ACC* anterior cingulate cortex, *CC* calcarine cortex, *dACC* dorsal anterior cingulate cortex, *DMN* default mode network, *dmPFC* dorso-medial prefrontal cortex, *EPP* emotion perception and processing, *ESM* experience sampling method, *FFA* fusiform face area, *FG* fusiform gyrus, *GFSS* Global Functioning Social Scale, *HC* healthy control participants, *IFG* inferior frontal gyrus, *INP* inpatient, *IRI* Interpersonal Reactivity Index, *l* left, *mPFC* medial prefrontal cortex, *MSCEIT* Mayer-Salovey-Caruso Emotional Intelligence Test, *n.r*. not reported, *NP-SZ* non-psychotic schizophrenia, *OFA* occipital face area, *OFC* orbitofrontal cortex, *P-SZ* psychotic schizophrenia, *PFC* prefrontal cortex, *PPI* psychophysiological interaction analysis, *QLS* Heinrichs-Carpenter Quality of Life Scale, *r* right, *ROI* region of interest, *SAS* Social Adjustment Scale, *SBM* Social behaviour in the milieu, *SF* social functioning, *SLOF* Specific levels of functioning, *SMA/aMCC* supplementary motor area/anterior midcingulate cortex, *SOFAS* Social and Occupational Functioning Assessment Scale, *SS* social skills, *STS* superior temporal sulcus, *TPJ* temporo-parietal junction, *ToM* Theory of mind, *vlPFC* ventro-lateral prefrontal cortex, *vmPFC* ventro-medial prefrontal cortex.

### Statistical analysis of the behavioural data

All analyses were carried out with STATA (version 17). Behavioural results were quantified in terms of correlations. Higher scores mostly reflected better SC performance and better social outcomes, however in some cases this association was reversed. All correlations were recoded so that positive correlations indicated associations between better SC performance and better social outcome. If a study reported several SC-outcome correlations within the same domain, correlations were averaged. All correlations were transformed with Fisher’s r-to-z transformation before meta-analysis. Results from the meta-analysis were back-transformed into raw correlation metric for presentation.

Meta-analyses on a SC domain were performed when at least two behavioural studies reported on the correlations between SC and a social outcome measure, resulting in 4 meta-analyses on the correlations: (i) between ToM and SF, (ii) ToM and SS, (iii) EPP and SF, and (iv) EPP and SS. We used a random-effects model to account for heterogeneity and to obtain unconditional inferences about the distribution of population correlations [54, 55]. The amount of heterogeneity in the true correlations was estimated with restricted maximum-likelihood estimation. For each of the individual meta-analyses, we report the number of studies, the estimated average correlation in the population distribution, CI (95% confidence interval for *ûp*), *p* (*p*-value for the test H0: *ûp* = 0), and the results from the Q-test for heterogeneity. Additional indices of the amount of variability in the correlations were Τ^2^ (estimated amount of heterogeneity in the true (transformed) correlations), H^2^ (total variability in the observed (transformed) correlation coefficients/within-study variance due to sampling error), and I^2^ (percentage of the total variability in the observed (transformed) correlation coefficients due to heterogeneity). A value of I^2^ close or equal to 0 suggests the absence of heterogeneity. Publication bias was examined through funnel plots, with the Fisher’s z on the x-axis and standard error on the y-axis. The Egger’s regression test was performed to investigate publication bias by asymmetry of the funnel plots.

## Results

The included studies comprised 1752 individuals and examined 24 associations between ToM and EPP and social outcomes. The average age of the study participants across the behavioural studies was 33.6 (range 22.4 to 45.3), 68.5% were male (range 50.0 to 86.7), 73.5% were white (range 54.3 to 97.4) and 82.4% had a diagnosis of schizophrenia (range 42.2 to 100). On average, study participants in included studies completed 12.6 years of education (range across studies 11.1 to 14).

### Theory of mind

**Fig. 1:**
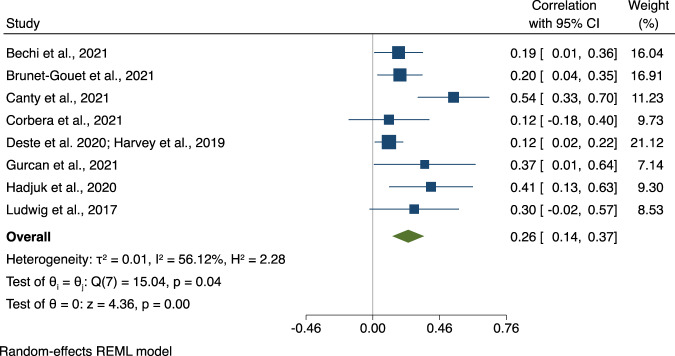
Association between Theory of Mind and Social Functioning. Note. Forest plot of individual effect sizes and overall association between Theory of Mind and Social Functioning. Horizontal lines represent 95% CIs. The area of each blue square is proportional to the study weight in the analysis. The green diamond represents pooled estimates from random-effects meta-analysis.

**Fig. 2:**
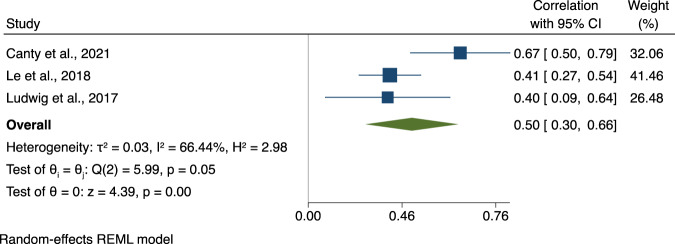
Association between Theory of Mind and Social Skills. Note. Forest plot of individual effect sizes and overall association between Theory of Mind and Social Skills. Horizontal lines represent 95% CIs. The area of each blue square is proportional to the study weight in the analysis. The green diamond represents pooled estimates from random-effects meta-analysis.

Since 2017, nine studies examined the cross-sectional associations between ToM and SF. The individual studies showed small to large effect size associations ranging from 0.12 to 0.54 (see Fig. 1). The overall effect size association was moderate (*ûp* = 0.26, CI95% [0.14, 0.37], *p* < 0.001). Three studies reported associations of 0.40 to 0.67 between ToM and SS (see Fig. 2), showing an average to large-sized association between the two domains (*ûp* = 0.50, CI95% [0.30, 0.66], *p* < 0.001).

### Meta-regression

Meta-regression analyses showed a significant negative association between duration of illness and the effect of studies exploring the association between ToM and SF, such that studies with samples with a shorter illness duration reported stronger effect sizes (*β* = -0.03, *z* = -2.59, CI95% [-0.05, -0.007], *p* = 0.01). Other meta-regression analyses did not show any significant associations.

### Publication bias

**Fig. 3:**
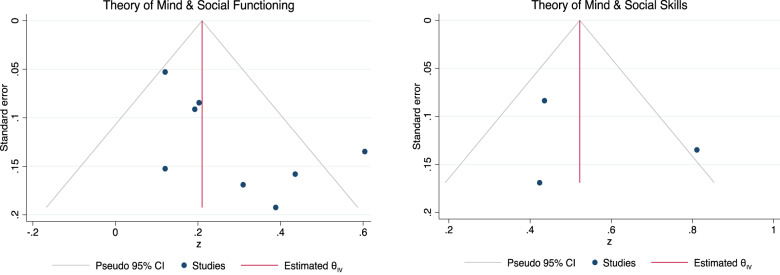
Funnel plot asymmetry of the associations between Theory of Mind, Social Functioning and Social Skills. Note. Visualisation of tests of funnel plot asymmetry for associations between Theory of Mind, Social Functioning (left panel) and Social Skills (right panel).

The Egger’s regression test for funnel plot asymmetry revealed some evidence for small-study effects in the meta-analysis exploring the association between ToM and SF (*β1* = 2.26, *se* = 0.83, *z* = 2.73, *p* = 0.006) but not between ToM and SS (*β1* = 0.77, *se* = 5.01. *z* = 0.15, *p* = 0.88; see Fig. 3 for the funnel plots). For the association between ToM and SF, we conducted a sensitivity analysis removing the study with outlying results by Canty and colleagues (2021) [56], resulting in an effect size reduction to a smaller overall effect size (*ûp* = 0.19, CI95% [0.11, 0.27]). After removing the study by Canty and colleagues (2021) [56] with outlying results for the association between ToM and SS the effect size reduced to medium to large (*ûp* = 0.43, CI95% [0.29, 0.58]).

### Emotion perception and processing

**Fig. 4:**
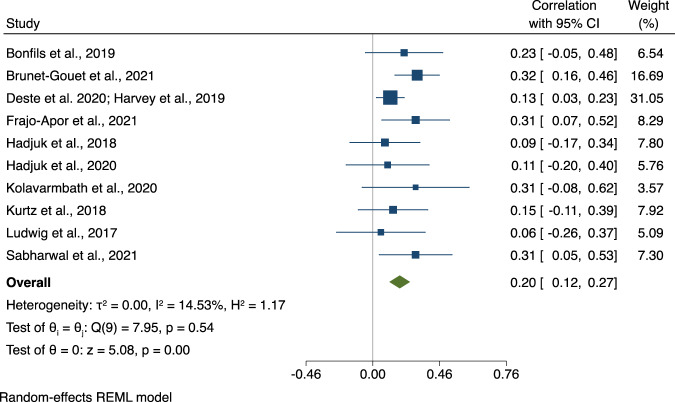
Association between Emotion Perception and Processing and Social Functioning. Note. Forest plot of individual effect sizes and overall association between Emotion Perception and Processing and Social Functioning. Horizontal lines represent 95% CIs. The area of each blue square is proportional to the study weight in the analysis. The green diamond represents pooled estimates from random-effects meta-analysis.

**Fig. 5:**
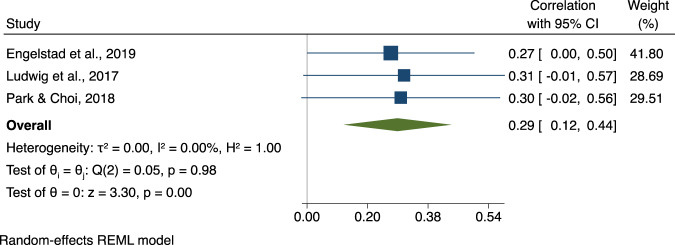
Association between Emotion Perception and Processing and Social Skills. Note. Forest plot of individual effect sizes and overall association between Emotion Perception and Processing and Social Skills. Horizontal lines represent 95% CIs. The area of each blue square is proportional to the study weight in the analysis. The green diamond represents pooled estimates from random-effects meta-analysis.

Thirteen studies investigated the association between EPP and social outcomes, of which 11 explored the association between EPP and SF and 3 between EPP and SS (one study investigating both). Meta-analysis results for EPP and SF are shown in Fig. 4. Studies had small to moderate associations ranging from 0.06 and 0.32 (*ûp* = 0.20, CI95% [0.12, 0.27], *p* < 0.001). The three studies on EPP and SS yielded a moderate-sized association (*ûp* = 0.29, CI95% [0.12, 0.44], *p* < 0.001; see Fig. 5).

### Meta-regression

Meta-regression analyses showed that study variation in age, sex, ethnicity, or illness duration did not moderate associations between EPP and SF significantly (range *p* = 0.09 to 0.98).

### Publication bias

**Fig. 6:**
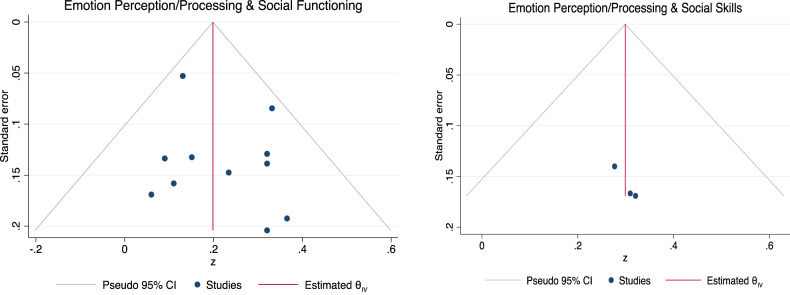
Funnel plot of the associations between Emotion Perception and Processing, Social Functioning and Social Skills. Note. Visualisation of tests of funnel plot asymmetry for the associations between Emotion Perception and Social Functioning (left panel) and Social Skills (right panel).

The Egger’s regression test for funnel plot asymmetry did not reveal any evidence for small-study effects in meta-analyses exploring the association between EPP and social outcomes (SF: *β1* = 0.51, *se* = 0.84, *z* = 0.61, *p* = 0.54; SS: *β1* = 1.38, *se* = 6.59, *z* = 0.21, *p* = 0.83). See Fig. 6 for the funnel plots.

#### Neuroimaging studies

We identified 14 studies that associated the neural correlates of SC task performance (five ToM, seven EPP) with SF (13) or SS (1). Two studies captured more complex SC, i.e., trust and trustworthiness, in simulated social interactions. The included studies comprised 285 patients and 284 healthy control participants. However, only two studies reported the demographic measures in healthy controls, and of these 30 healthy control participants data are presented below. The average age of the patients across the neuroimaging studies was 34.9 (range 15 to 65 years), and 30.3 (range 18 to 65 years) for healthy controls. Of the patients, 69.5% (range 48 to 100) were male (80.0% for healthy controls, range 66.7- to 100), 60.7% were white (range 43 to 83, data missing for healthy controls). On average, patients completed 13.5 (range 11.9 to 15) years of education, and healthy controls 15.3 (range 14.2 to 16.9). Duration of illness was 13.9 (range < 2 to 19.5) years and 83.8% had a diagnosis of schizophrenia.

#### Theory of mind

Of the five studies on ToM, three adopted a whole brain approach [51, 57, 58], and two used predefined regions of interest [59, 60]. While two studies [57, 60] did not find any significant associations between neural activation in mainly prefrontal and parietal social brain regions during a ToM task and SF (*r*’s not reported), the other two [58, 59] found moderate to large correlations between mPFC activation during ToM and better SF (*r’s* = 0.44 and 0.54). In contrast, one study [51] found a large correlation between increased IFG activation and worse SF (*r* = -0.53).

Lee et al. [58] adopted a longitudinal approach. Participants were scanned twice, once during an acute psychotic episode that was severe enough to warrant inpatient admission, and once close to being discharged from the inpatient unit. ToM performance and SF improved, and neural activation in the left mPFC, the right fusiform gyrus, the posterior middle temporal, and lingual gyri, and in the left IPL was increased at discharge as compared to before. However, only increased activation of the left mPFC was marginally significantly correlated with improvement in SF [58].

Dodell-Feder and colleagues [59] additionally used a whole brain analysis and also investigated brain activation-social functioning associations in healthy participants, allowing for group comparison to SZ. Associations between neural activation during ToM with SF were apparent in the same brain regions in individuals diagnosed with SZ and healthy controls. That is, across all participants greater mPFC activity during a false belief task compared to a control condition was associated with better SF. Furthermore, in both groups rTPJ activity was also correlated with social behaviour in the milieu (managing emotions). However, some differences emerged where in SZ only the association between mPFC and SF as measured by the Social Adjustment Scale, but not the Global Functioning: Social Scale, was significant, while in controls only the association between mPFC and SF as assessed by Global Functioning: Social Scale was significant. The study supports the importance of ToM-related neural circuitry for social functioning, although further investigations will be needed to clarify the heterogenous findings related to the different outcome measures [59].

Associations in the opposite direction were reported by Das et al. [51] who showed that increased activation in the IFG during intention recognition as compared to random movement of triangles, was associated with reduced SF. Brain areas that showed significant group differences were identified in two ToM related areas, i.e., in the IFG and TPJ (STG), where patients showed reduced activation compared to controls. In patients, these activations were associated with the LSP, showing negative associations. Although the association between SLF and STS was not significant, the association between STS activation and IFG was, indirectly suggesting a potential meaningful relationship [51]. In sum, the results of studies examining the association between ToM-related brain activity and social outcomes are conflicting in terms of the associations and implicated brain areas. However, two independent studies show a positive association between ToM-related brain activation, specifically in the mPFC and SF, again pointing towards an important role of this area. The IFG, and relatedly the STS, associated with more implicit, automatic ToM processes, appear to impact differently on SF, given the opposite, negative association [51]. This may suggest that higher task-related activation in these areas reflects different mechanisms, for example more efficient processing vs. greater processing effort.

#### Emotion perception and processing

Five EPP studies used a region of interest-based approach [61–65] and three investigated whole brain activation [65–67]. In both types of studies, reported correlations pointed to moderate to strong associations of neural activation during EPP with SF (*n* = 7) and social behaviour in the milieu (*n* = 1) (range *r* = 0.44-69).

One study showed that signal changes in the amygdala in response to direct-gaze anger was strongly correlated with SF (*r* = 0.58) [63]. Two other region of interest studies from the same dataset showed patterns whereby higher brain activation and connectivity during EPP was associated with better SF. Specifically, Nelson et al. [62] reported an association between decreased ACC activation in response to pleasant images (compared to a control condition) and poorer SF, and Bjorkquist et al. [61] found that weaker connectivity between the amygdala and right mPFC during EPP (viewing negative vs. neutral face images) was related to poorer SF. However, after Bonferroni correction, this finding only approached significance. One study reported a reverse pattern with greater neural activation in the IFG, insula and calcarine cortex during EPP (emotion vs. neutral face viewing) being associated with worse SF. Also, higher left amygdala-IFG, and left amygdala-insula functional connectivity were found to be negatively associated with SF [64].

The three whole brain studies varied in complexity. Taylor and colleagues [65] required appraisal of faces (“likeable or unlikeable?”), Smith and colleagues [66] used a paradigm during which participants had to choose the appropriate emotional expression to a situation, and Shin and colleagues [67] used a virtual reality (VR) task, in which participants determined whether or not the avatar’s speech was appropriate in a given situation. Taylor et al. [65] found that increased occipital lobe activity correlated with poorer SF. During Smith et al.’s [66] emotion selection task activation in the right superior motor area (SMA), extending to the anterior midcingulate cortex (aMCC) was positively correlated with SF and SS, i.e., social attainment and competence, and activation in the right precuneal sulcus correlated with social attainment at trend level. Exploratory analyses with activation clusters outside hypothesised regions showed an additional positive association between right cuneus activity and SF (social competence). Finally, Shin et al. [67] reported that neural activation of the dlPFC, but not of the STS, was positively correlated with SF and argued that dlPFC hypoactivity-related cognitive inflexibility may underpin social dysfunction in schizophrenia [67].

**Fig. 7:**
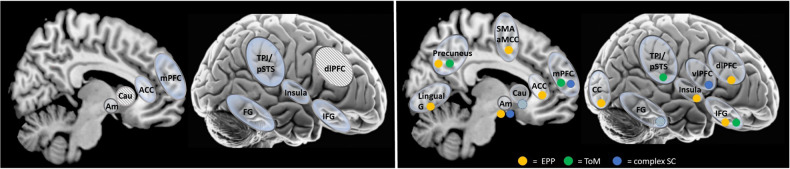
Social Brain Network (a), and Areas Associated with Social Outcomes in SZ (b). **a** Dashed brain regions represent brain regions associated with social decision making that are not usually viewed as components of the social brain; **b** coloured dots indicate the association of the activation of the area with social functioning (yellow = EPP, green = ToM, blue = complex SC); dashed coloured dots represent associations at trend level. Please note the dots do not represent exact coordinates of the study ROIs. ACC anterior cingulate cortex, Am amygdala, Cau caudate, CC calcarine cortex, dlPFC dorsolateral prefrontal cortex, FG fusiform gyrus, G gyrus, IFG inferior frontal gyrus, mPFC medial prefrontal cortex, TPJ/pSTS temporo-parietal junction/posterior superior temporal sulcus, vlPFC ventrolateral prefrontal cortex.

Overall, the EPP findings revealed mostly, although not exclusively, moderate to strong positive associations between brain activation during EPP tasks and better SF and SS in SZ. Regions that correlated with social outcomes were located both in- and outside the social brain (see Fig. 7). Region of interest studies reported predominantly associations between better social outcomes and activation in selected regions in the frontal cortex and amygdala. Studies adopting a whole brain approach also reported associations between social outcomes and activation in mid-frontal, occipital and subcortical brain regions.

#### Complex social cognition

Two studies investigated associations between trust and trustworthiness and SF, reporting small to medium effect sizes [68]. Brain regions showing significant associations varied widely. In one of these studies, Pinkham et al. [69] asked paranoid and non-paranoid SZ participants to rate whether a face was trustworthy or not. In all participants, increased activation in response to untrustworthy compared to trustworthy faces in the bilateral fusiform gyrus, mPFC, and bilateral ventrolateral prefrontal cortex (vlPFC) was associated with better SF. The study also showed that in healthy controls, this association with SF was apparent for the right amygdala, bilateral fusiform gyrus, mPFC, and right vlPFC. In non-paranoid patients, these associations were not significant and in the paranoid group, the only significant correlation with SF was found for the left amygdala. A marginally significant association was found between SF and the right FG. The second study used an interactive trust game paradigm, participants made decisions on whether to trust the counterpart. Participants were given some information on the degree of their trustworthiness (low/high) or received no prior information. Lower perceived social exclusion, as measured by experience sampling, was marginally associated with higher right caudate activation in the high trustworthiness context only. Here, higher perceived social relationship quality was also associated with greater mPFC activation. The amount of time people spent alone or in company, the emotional responses to the company and feelings of loneliness did not show significant correlations with neural activation patterns. Despite the differences in methodology and outcome, both studies showed increased SF with increased brain activation, and an overlap of activation in the mPFC.

## Discussion

Individuals with a diagnosis of SZ often experience major social impairments across the lifespan, but the underlying cognitive and neural processes are still not fully understood [70]. This review and meta-analysis provide an overview of the most recent findings on the relationship between different SC domains, their neural substrates, and different types of social outcomes; extending the evidence from previous meta-analyses that focused predominantly on the relationships between SC and the broader outcome of community functioning in schizophrenia [5, 6].

### Social cognition and outcomes

Most studies focused on the domains ToM and EPP in relation to SF in terms of social outcome, which were largely based on questionnaire-based assessments of the number of close friends, or the frequency of social contacts. Fewer studies focused on social behaviour in the milieu or SS-based outcomes, such as the capacity to perform social behaviours in structured settings, for example in role plays [71]. We did not identify sufficient behavioural or neuroimaging studies that investigated associations between social perception and bias, attribution bias, or overall SC and social outcomes for meta-analyses.

#### Theory of mind

While previous research observed the strongest associations between ToM and broad indicators of community functioning [5], which comprised social functioning but also non-social aspects such as independent living skills, the current meta-analysis of three more recent studies showed the strongest association between ToM and SS (overall *ûp* = 0.50). In contrast, the associations between ToM and SF were in the small to moderate range (overall *ûp* = 0.26). This finding is more in line with the recent meta-analysis by Halverson and colleagues [6], who also showed stronger associations for three studies on ToM and SS vs. 25 studies that looked at ToM and community functioning (*ûp* = 0.36 vs. *ûp* = 0.21, respectively). However, it remains to be seen whether this pattern still holds in future replication studies. Our meta-regression analyses showed that the association between ToM and SF was strongest for studies including individuals with a shorter illness duration, possibly highlighting the importance of early interventions when it comes to beneficial effects of ToM interventions on SF.

Neuroimaging studies mostly examined ToM-related neural activation in relation to aspects of community SF. The findings of the five available studies on associations between ToM-related brain activation and different social outcomes were mixed, with two studies showing that in patients increased neural activation in the mPFC was associated with better SF on some, but not all, social outcome measures [62, 59]. One study showed an association between increased activation of the IFG and worse SF [51], and two studies did not show any significant associations between neural activation during ToM and SF [57, 60]. While the mPFC might be particularly important for the relationship between ToM and SF, due to the scarcity of findings, variety of utilised paradigms and analysis approaches in five small samples, no firm conclusions can as yet be drawn about the neural substrates that underlie the ToM-social outcome link.

#### Emotion perception and processing

For EPP we found moderate effect size associations that were stronger for SS (*ûp* = 0.29) than for SF (*ûp* = 0.20) although also here only three behavioural studies on EPP and SS could be included. A slightly lower effect size for EPP and SS (*ûp* = 0.25), yet stronger effect size than for EPP and community functioning (*ûp* = 0.22) was reported by Halverson and colleagues [6], based on 10 studies. Our meta-regressions did not reveal any relevant moderators.

Neuroimaging studies reported almost exclusively on associations between EPP and SF. ROIs were mostly focused on the amygdala although some studies also included mPFC, IFG, ACC, insula, and occipital regions. Five studies showed that in patients, higher neural activation in amygdala, mPFC, ACC and dlPFC or increased functional connectivity between amygdala and mPFC during EPP was associated with better SF [61–63, 66, 67]. In contrast, in two other studies, increased neural activation during EPP correlated with poorer SF. Taylor and Chen [65] showed that occipital activation was associated with poorer SF and therefore suggested that early visual processing may play a role in SF. Sabharwal et al. [64] showed that increased amygdala connectivity with insula and IFG was associated with worse SF. They suggested that the counterintuitive finding may indicate inefficient processing which leads to upregulation of emotion processing (i.e., effort).

Some variation in findings could be explained by the nature of the different EPP tasks. That is, while the majority of studies used fairly comparable paradigms, which included images of faces expressing emotions that had to be appraised [65], identified [63], matched [64] or rated for intensity [61, 62], one study presented a task during which a proper emotional response had to be rated and selected for a specific social situation [66], while another task asked the participant to determine the emotion of one of the two interacting partners. In the latter two studies, increased activation outside the typical social brain areas (i.e., SMA, aMCC, and lingual gyrus) was associated with better SF, highlighting the involvement of other cognitive functions on social outcomes.

#### Complex social cognition

As for ToM and EPP, available evidence on complex SC also showed that increased neural activation in prefrontal areas and the amygdala was associated with better SF and higher reported relationship quality. Moreover, findings on higher caudate activation during experiences of trust and benevolent social interactions (i.e., repayments in a trust game), may reflect the experience of social reward and therefore relate to a higher sense of belonging or inclusion in social relationships. Social reward could activate SC processes. Concluding, the two available studies support the idea that neural modulation during complex SC has potential as a predictive marker of real-world social behaviour, however further research on the link between complex SC and its relationship to more basic SC function is needed [68, 69].

#### Social cognition and social outcome - what do we know about the association?

Supportive of the NIMH workshop goals to establish the significance of SC in SZ [14], various studies showed small to moderate effect size associations between SC and real-life community or social functioning, or indices for the capacity thereof [5, 6]. While these studies cannot evidence causality, a causal relationship between SC and social outcomes has been suggested by promising results of interventions for SC that resulted in improvements in social outcomes [72–75]. However, enthusiasm is somewhat hampered by methodological issues, such as the quality of SC tests, small samples sizes, non-blinded assessments, or the lack of well-controlled Randomised Controlled Trials.

Two key questions remain: 1) are there any specific SC functions that are particularly important for social outcome and 2) are there specific social outcomes that are more strongly associated with aspects of SC than others. We found the strongest associations between ToM and SS followed by EPP and SS and somewhat lower associations between both ToM and EPP and SF, which is in line with previous reports [6] and indicative of the greater role of SC in more proximal, skills-based measures. These measures indicate the capacity for social functioning under optimal conditions and might be less confounded by other factors, such as lack of resources or an already collapsed social network, that might impede social functioning in the real world.

It seems self-evident that different SC functions are necessary to enable humans to interact effectively with the social world and that reduced SC ability therefore leads to social misperceptions, misinterpretation and interpersonal problems. Couture et al. (2007) proposed a conceptual model of SC and social outcomes, according to which SC impairments may lead to poorer social outcomes through a cascade of related mechanisms, beginning with the aberrant perception and interpretation of “noisy” social data from the environment, that results in suspiciousness, felt rejection or exclusion, and consequently may lead to negative social interactions or avoidance of social interaction and social isolation [76]. However, the underlying mechanisms and intermediary factors by which SC and social outcomes are connected are less well researched. In future, it will be important that researchers test this model. Other factors that have been associated with poorer SC and that could worsen social outcomes include, among others, negative symptoms, social competency [77], defeatist beliefs, self-stigma, and the ability to generate social support, all of which may reduce the motivation and opportunity to engage in social interaction should be investigated in this context [78–80]. SC has also been linked to attachment [81], which might explain an individual’s fundamental beliefs about the safety in social interactions and the own willingness and ability to engage in social interactions.

One underlying rationale of SC interventions is that they lead to neurobiological changes and “social brain” plasticity, that translate into improvements in SC and subsequently improved social functioning [82, 83]. Evidence from five neuroimaging studies suggests that SC interventions may normalise, that is, increase regional brain activity (e.g., in left MFG, IPL, STG, hippocampus, amygdala, etc.) [84] and ToM and EPP training effects on mPFC activation have been shown to predict real world functioning [85]. While the implicated brain regions have been inconsistent across studies, these findings suggest that neuroimaging can provide insights into the shared and distinct underlying mechanisms that mediate SC-social outcome associations.

#### What have we learnt from neuroimaging studies on social cognition and social outcomes?

In general, individuals with a diagnosis of schizophrenia perform worse than controls on SC MRI tasks, and show reduced neural activation, and poorer social functioning [21, 22]. Most of the fMRI studies that we have reviewed here showed that increased neural activation in various ToM, EPP and complex SC tasks vs. a control condition is associated with better social outcomes in the real-world. One study also showed this for role play assessment of social competence. The available studies were cross-sectional and cannot speak to the directionality of effects. The reviewed studies suggest that it is possible that reduced neural activation in response to social cues may lead to less engagement of the mentalising, emotion processing and reward networks and therefore poorer social outcomes. However, vice versa, it is possible that poor social functioning, social withdrawal, and deprivation, which occur already before the first psychotic episode in SZ [1, 2, 45, 70], might impact brain structure and function [86–88, 72–74] so that altered neural activation patterns during SC tasks may be a result. One study showed that higher activation of the rostral lateral prefrontal cortex during social cognitive introspective accuracy was associated with better social functioning, as measured with the Specific Level of Functioning Scale [89].

Interestingly, while behavioural findings suggest that ToM and EPP may be more strongly related to SS than SF and with stronger associations for ToM than EPP overall, the neuroimaging studies tentatively suggest that the effect sizes for correlations between neural activation in various regions during task performance within both SC domains and SF are similar [90]. The studies in this review showed some overlap in the brain regions that were activated during ToM and EPP and SF. Notably, these regions were the precuneus and IFG for EPP, ToM and SF, the amygdala for EPP, complex SC and SF, and the mPFC for ToM, complex SC and SF, suggesting that these regions might be useful shared targets for treatment, for example for neurostimulation in attempts to improve SF. However, so far, no brain regions showed associations with SF for all three SC domains.

The heterogeneity in the neuroimaging findings may be due to heterogeneity in approaches, including, among others, variation in measures, fMRI set-up and analysis, and makes it difficult to draw firm conclusions. Social outcome measures for SF were mostly broad, measuring social adjustment, quality of life, level of functioning, social and in some cases occupational functioning. The Heinrichs-Carpenter Quality of Life Scale was the only scale that was used in more than one imaging study (*n* = 3). The findings of the studies were heterogenous with one reporting no significant association with brain activation during ToM, one reporting a positive association between higher ACC activation and better SF and one reporting a negative association in the IFG, calcarine sulcus and insula during EPP and SF. Associations with social functioning were mostly reported and investigated in the patient group only. The majority of studies (10) investigated the association with social functioning in regions where a significant group difference with healthy controls was found. Four studies investigated the association in all regions of interest, regardless of group differences, of which one study only looked in patients [64]. The other three studies showed results across all participants, and between groups, in regions that did not always show group differences [59, 60, 69]. The meaning of these associations remains to be investigated. Together, neuroimaging studies have yet to explore the neural association between SC and the more proximal, performance-based aspects of social outcomes.

One important limitation of existing neuroimaging studies lies in their narrow, ROI-based approach of single key-regions that have been associated with specific SC functions. This may overlook important other regions and precludes investigation of shared and distinct neural processes that may underlie the SC relationship with different aspects of social functioning [14]. Another approach to selecting ROI was to focus on regions that showed significant differences between individuals with SZ and a control group, to uncover whether significant group differences in activation might explain some of the deficits in SF that are observed in patients compared to controls. Following the approach of Dodell-Feder et al. [59] and Smith et al. [66], it can be informative to also study associations between social functioning and neural activation during SC in controls and first-degree relatives of individuals with psychosis with an elevated genetic risk to understand better how the social brain facilitates the SC and SF link in health and psychosis.

#### Directions for future research

**Fig. 8:**
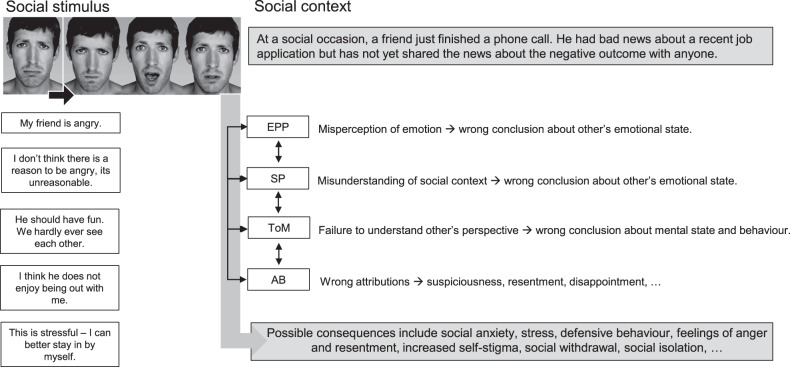
Pathways between social environment, social cognition, and social outcome. Note: Image: Adapted to show four emotions from Stuart Hamilton (2005, 5 emotions, https://www.flickr.com/photos/stuandgravy/4032861, CC BY-NC-SA). AB attriutional bias, EPP emotion perception and processing, SP social perception, ToM, theory of mind.

The current study highlights the need for further research in several areas. First, many studies still focus on single SC domains, mostly EPP and ToM, and on selected social outcomes, which are often assessed with single measures. To allow for comparison of specific associations between SC domains and different social outcomes more comprehensive measurement will be important. Data-pooling initiatives and special interest consortia will be fruitful avenues to generate more comprehensive and comparative insights. Second, studies generally investigated SC by means of paradigms that require the interpretation of social stimuli in stories, cartoons or pictures and used questionnaires to measure SF, while initial studies started to employ more complex and social interactive tasks that tap into different SC mechanisms and/or assessed real-world social functioning using experience sampling, there is a greater need for an ecologically valid assessment. Social exchange paradigms that simulate social interaction in a controlled setting may be one way forward in the endeavour [91]. Third, there is some evidence suggesting that accurate insight into the own SC ability and task performance [89] and informant-based reports of SC ability may be much better predictors of real-world social functioning than actual SC test performance [47]. While the latter may not always be available to researchers or clinicians, assessing introspective accuracy and social metacognition might be a viable and fruitful strategy in the prediction of social outcomes, which should be explored further by future research. Fourth, the pathways by which SC impairment may lead to poorer social outcomes (Fig. 8) have been theorised about, but empirical investigations of these pathways are still scarce. Fifth, few neuroimaging studies focused on the investigation of the SC – social outcome link. Studies that directly compare the neural mechanisms that underlie the associations between different SC domains – and different social outcome domains using neuroimaging might further elucidate shared or unique mechanisms for the domains. Future studies should attempt analysis of wider social brain structure and/or exploratory whole brain analysis and study those mechanisms in controls, as well as individuals across the entire spectrum of psychotic disorders and those with an increased familial risk for psychosis to generate broader insights. Previous work suggested that cognition and brain function are related to changes in brain structure [45], but more evidence is needed that investigate the structure-functioning link with respect to SC and social outcomes. Finally, in several studies null-findings were not reported. These should be included in future research.

## Conclusions

This systematic review and meta-analysis described the knowledge base on different SC mechanisms and social outcomes and the underlying neural mechanisms of these. Overall, the findings showed that ToM and EPP were more strongly associated with more proximal measures of social capacity than measures that capture real-world social functioning. Functional neuroimaging studies on the association were still scarce and mostly focused on EPP and SF. Most of the initial evidence points towards patterns whereby higher brain activation in regions of the social brain during SC is associated with better social outcomes. However, future research will need to show whether these patterns hold and are comparable across SC functions and outcomes.

## References

[1] Velthorst E (2016). Developmental Trajectories of Impaired Community Functioning in Schizophrenia. JAMA Psych.

[2] Velthorst E (2017). The 20-year longitudinal trajectories of social functioning in individuals with psychotic disorders. Am J Psychiatry.

[3] Arango C (2011). Psychopathology, coronary heart disease and metabolic syndrome in schizophrenia spectrum patients with deficit versus non-deficit schizophrenia: findings from the CLAMORS study. Eur Neuropsychopharmacol.

[4] Wang J (2017). Social isolation in mental health: a conceptual and methodological review. Soc Psychiatry Psychiatr Epidemiol.

[5] Fett AK (2011). The relationship between neurocognition and social cognition with functional outcomes in schizophrenia: a meta-analysis. Neurosci Biobehav Rev.

[6] Halverson TF (2019). Pathways to functional outcomes in schizophrenia spectrum disorders: Meta-analysis of social cognitive and neurocognitive predictors. Neurosci Biobehav Rev.

[7] Javed, A and A Charles, The Importance of Social Cognition in Improving Functional Outcomes in Schizophrenia. Front Psychiatry, 2018;9:157. 10.3389/fpsyt.2018.00157 PMC5928350 29740360

[8] Savla GN (2013). Deficits in domains of social cognition in schizophrenia: a meta-analysis of the empirical evidence. Schizophrenia Bull.

[9] Georgiades A (2017). Psychometric characteristics of the MATRICS Consensus Cognitive Battery in a large pooled cohort of stable schizophrenia patients. Schizophr Res.

[10] Fett A-KJ, Reichenberg A, Velthorst E (2022). Lifespan evolution of neurocognitive impairment in schizophrenia - A narrative review. Schizophrenia Res.: Cognition.

[11] Gold JM (2004). Cognitive deficits as treatment targets in schizophrenia. Schizophrenia Res.

[12] Pinkham AE, Harvey PD, Penn DL (2017). Social Cognition Psychometric Evaluation: Results of the Final Validation Study. Schizophrenia Bull.

[13] Pinkham AE (2014). The social cognition psychometric evaluation study: results of the expert survey and RAND panel. Schizophrenia Bull.

[14] Green MF (2008). Social cognition in schizophrenia: an NIMH workshop on definitions, assessment, and research opportunities. Schizophrenia Bull.

[15] Wojtalik JA (2017). A Systematic and Meta-analytic Review of Neural Correlates of Functional Outcome in Schizophrenia. Schizophrenia Bull.

[16] Blakemore S-J (2008). The social brain in adolescence. Nat Rev Neurosci.

[17] Barton RA, Dunbar RI (1997). Evolution of the social brain. Machiavellian intell II: Ext Eval.

[18] Dunbar RI, Shultz S (2007). Evolution in the social brain. science.

[19] Meyer ML (2019). Social by Default: Characterizing the Social Functions of the Resting Brain. Curr Dir Psychol Sci.

[20] Pinkham, AE, Social cognition in schizophrenia. J Clin Psych, 2014;75:14–9. 10.4088/JCP.13065su1.04 24919166

[21] Fett A-K, Shergill S, Krabbendam L (2015). Social neuroscience in psychiatry: unravelling the neural mechanisms of social dysfunction. Psychol Med.

[22] Sugranyes G (2011). Autism spectrum disorders and schizophrenia: meta-analysis of the neural correlates of social cognition. PloS one.

[23] Kronbichler L (2017). Abnormal brain activation during theory of mind tasks in schizophrenia: a meta-analysis. Schizophrenia Bull.

[24] Weng Y, et al. Neuropathways of Theory of Mind in Schizophrenia: A Systematic Review and Meta-Analysis. Neurosci Biobehav Rev, 2022;137:104625. 10.1016/j.neubiorev.2022.104625 35339482

[25] Vucurovic K, Caillies S, Kaladjian A. Neural Correlates of Mentalizing in Individuals With Clinical High Risk for Schizophrenia: ALE Meta-Analysis. Front psych, 2021;12:634015. 10.3389/fpsyt.2021.634015 PMC8095711 33959048

[26] Vucurovic K, Caillies S, Kaladjian A (2020). Neural correlates of theory of mind and empathy in schizophrenia: An activation likelihood estimation meta-analysis. J Psychiatric Res.

[27] Jáni M, Kašpárek T (2018). Emotion recognition and theory of mind in schizophrenia: a meta-analysis of neuroimaging studies. World J Biol Psychiatry.

[28] Li H (2010). Facial emotion processing in schizophrenia: a meta-analysis of functional neuroimaging data. Schizophrenia Bull.

[29] Taylor SF (2012). Meta-analysis of functional neuroimaging studies of emotion perception and experience in schizophrenia. Biol Psychiatry.

[30] Lukow P (2021). Neural correlates of emotional processing in psychosis risk and onset–A systematic review and meta-analysis of fMRI studies. Neurosci Biobehav Rev.

[31] Dong D (2018). Abnormal brain activation during threatening face processing in schizophrenia: a meta-analysis of functional neuroimaging studies. Schizophrenia Res.

[32] Baas D (2008). Social judgement in clinically stable patients with schizophrenia and healthy relatives: behavioural evidence of social brain dysfunction. Psychological Med.

[33] Brüne M (2005). “Theory of mind” in schizophrenia: a review of the literature. Schizophrenia Bull.

[34] Horat SK (2018). Impaired social cognition in schizophrenia during the Ultimatum Game: an EEG study. Schizophrenia Res.

[35] Billeke P (2015). Paradoxical expectation: oscillatory brain activity reveals social interaction impairment in schizophrenia. Biol psychiatry.

[36] Brosnan MB, Wiegand I (2017). The Dorsolateral Prefrontal Cortex, a Dynamic Cortical Area to Enhance Top-Down Attentional Control. J Neurosci.

[37] Florio TM (2018). The Basal Ganglia: More than just a switching device. CNS Neurosci Therapeutics.

[38] Fett A-KJ (2019). The neural mechanisms of social reward in early psychosis. Soc Cogn Affect Neurosci.

[39] Gromann, P, et al., Trust versus paranoia: abnormal response to social reward in psychotic illness. Brain, 2013:awt076. 10.1093/brain/awt076 23611807

[40] Tschacher W, Giersch A, Friston K (2017). Embodiment and schizophrenia: a review of implications and applications. Schizophrenia Bull.

[41] Burns J (2006). The social brain hypothesis of schizophrenia. World Psych.

[42] Long M (2022). A systematic review of social functioning outcome measures in schizophrenia with a focus on suitability for intervention research. Schizophrenia Res.

[43] Buchanan RW (2021). Combined Oxytocin and Cognitive Behavioral Social Skills Training for Social Function in People With Schizophrenia. J Clin Psychopharmacol.

[44] Harvey PD, Isner EC (2020). Cognition, Social Cognition, and Functional Capacity in Early-Onset Schizophrenia. Child Adolesc Psychiatr Clin N Am.

[45] Valaparla VL (2021). Social cognitive deficits in schizophrenia and their neurocognitive correlates across the different phases of illness. Asian J Psychiatr.

[46] Mucci A (2021). Factors Associated With Real-Life Functioning in Persons With Schizophrenia in a 4-Year Follow-up Study of the Italian Network for Research on Psychoses. JAMA Psychiatry.

[47] Silberstein JM (2018). Self-assessment of social cognitive ability in schizophrenia: Association with social cognitive test performance, informant assessments of social cognitive ability, and everyday outcomes. Schizophr Res.

[48] Ludwig KA (2020). Correlates of loneliness among persons with psychotic disorders. Soc Psychiatry Psychiatr Epidemiol.

[49] Deste G (2020). Autistic Symptoms and Social Cognition Predict Real-World Outcomes in Patients With Schizophrenia. Front Psychiatry.

[50] Harvey PD (2019). Predictors of social functioning in patients with higher and lower levels of reduced emotional experience: Social cognition, social competence, and symptom severity. Schizophr Res.

[51] Das P (2012). Mentalizing impairment in schizophrenia: a functional MRI study. Schizophr Res.

[52] Thakkar KN, Peterman JS, Park S (2014). Altered brain activation during action imitation and observation in schizophrenia: a translational approach to investigating social dysfunction in schizophrenia. Am J Psychiatry.

[53] Csukly G (2016). Deficits in low beta desynchronization reflect impaired emotional processing in schizophrenia. Schizophr Res.

[54] Hedges LV, Vevea JL (1998). Fixed-and random-effects models in meta-analysis. Psychological Methods.

[55] Leucht S, Kissling W, Davis J (2009). How to read and understand and use systematic reviews and meta‐analyses. Acta Psychiatr Scandinavica.

[56] Canty AL (2021). The functional significance of cognitive empathy and theory of mind in early and chronic schizophrenia. Psychiatry Res.

[57] Bartholomeusz CF (2018). An fMRI study of theory of mind in individuals with first episode psychosis. Psychiatry Res Neuroimaging.

[58] Lee KH (2006). A functional magnetic resonance imaging study of social cognition in schizophrenia during an acute episode and after recovery. Am J Psychiatry.

[59] Dodell-Feder D (2014). The neural basis of theory of mind and its relationship to social functioning and social anhedonia in individuals with schizophrenia. Neuroimage Clin.

[60] Hyatt CJ (2020). Default mode network modulation by mentalizing in young adults with autism spectrum disorder or schizophrenia. Neuroimage Clin.

[61] Bjorkquist OA (2016). Altered amygdala-prefrontal connectivity during emotion perception in schizophrenia. Schizophr Res.

[62] Nelson BD (2015). Schizophrenia symptom and functional correlates of anterior cingulate cortex activation to emotion stimuli: An fMRI investigation. Psychiatry Res.

[63] Pinkham AE (2011). Abnormal modulation of amygdala activity in schizophrenia in response to direct- and averted-gaze threat-related facial expressions. Am J Psychiatry.

[64] Sabharwal A, Kotov R, Mohanty A (2021). Amygdala connectivity during emotional face perception in psychotic disorders. Schizophr Res.

[65] Taylor SF (2011). Social appraisal in chronic psychosis: role of medial frontal and occipital networks. J Psychiatr Res.

[66] Smith MJ (2015). Alterations in brain activation during cognitive empathy are related to social functioning in schizophrenia. Schizophr Bull.

[67] Shin JE (2015). Involvement of the dorsolateral prefrontal cortex and superior temporal sulcus in impaired social perception in schizophrenia. Prog Neuro-Psychopharmacol Biol Psychiatry.

[68] Hanssen E (2022). Neural, behavioural and real-life correlates of social context sensitivity and social reward learning during interpersonal interactions in the schizophrenia spectrum. Aust N Z J Psychiatry.

[69] Pinkham AE (2008). An investigation of the relationship between activation of a social cognitive neural network and social functioning. Schizophr Bull.

[70] Velthorst E (2019). Neurocognitive profiles in the prodrome to psychosis in NAPLS-1. Schizophr Res.

[71] Green MF, Llerena K, Kern RS (2015). The “Right Stuff” Revisited: What Have We Learned About the Determinants of Daily Functioning in Schizophrenia?. Schizophrenia Bull.

[72] Horan WP, Green MF (2019). Treatment of social cognition in schizophrenia: Current status and future directions. Schizophrenia Res.

[73] Kurtz MM, Richardson CL (2011). Social Cognitive Training for Schizophrenia: A Meta-Analytic Investigation of Controlled Research. Schizophrenia Bull.

[74] Vass E (2018). Interventions for the treatment of theory of mind deficits in schizophrenia: Systematic literature review. Psychiatry Res.

[75] Grant N (2017). Social cognition interventions for people with schizophrenia: a systematic review focussing on methodological quality and intervention modality. Clin Psychol Rev.

[76] Couture SM, Penn DL, Roberts DL (2006). The functional significance of social cognition in schizophrenia: a review. Schizophrenia Bull.

[77] Kalin M (2015). Social cognition, social competence, negative symptoms and social outcomes: Inter-relationships in people with schizophrenia. J Psychiatr Res.

[78] Green MF (2019). From Social Cognition to Negative Symptoms in Schizophrenia: How Do We Get There From Here?. Schizophrenia Bull.

[79] Costa-Cordella, S, et al., Social Support and Cognition: A Systematic Review. Front Psychol, 2021;12:637060. 10.3389/fpsyg.2021.637060 PMC7941073 33708164

[80] Turner DT (2017). A Meta-Analysis of Social Skills Training and Related Interventions for Psychosis. Schizophrenia Bull.

[81] Varela LF (2021). Attachment styles moderate Theory of Mind differences between persons with schizophrenia, first‐degree relatives and controls. Br J Clin Psychol.

[82] Tripathi A, Kar SK, Shukla R (2018). Cognitive Deficits in Schizophrenia: Understanding the Biological Correlates and Remediation Strategies. Clin Psychopharmacology Neurosci.

[83] Dodell-Feder D, Tully LM, Hooker CI (2015). Social impairment in schizophrenia: new approaches for treating a persistent problem. Curr Opin Psychiatry.

[84] Campos C (2016). Neuroplastic changes following social cognition training in schizophrenia: a systematic review. Neuropsychol Rev.

[85] Subramaniam K (2012). Computerized Cognitive Training Restores Neural Activity within the Reality Monitoring Network in Schizophrenia. Neuron.

[86] Hall FS (1998). Social deprivation of neonatal, adolescent, and adult rats has distinct neurochemical and behavioral consequences. Crit Rev™ Neurobiol.

[87] Orben A, Tomova L, Blakemore S-J (2020). The effects of social deprivation on adolescent development and mental health. The Lancet Child Adolesc Health.

[88] Sandi C, Haller J (2015). Stress and the social brain: behavioural effects and neurobiological mechanisms. Nat Rev Neurosci.

[89] Pinkham AE (2018). Neural correlates of social cognitive introspective accuracy in schizophrenia. Schizophr Res.

[90] Van Hooren S (2008). Social cognition and neurocognition as independent domains in psychosis. Schizophrenia Res.

[91] Fett A-KJ (2014). Trust and social reciprocity in adolescence–a matter of perspective-taking. J Adolesc.

[92] Bechi M, et al. Theory of mind and stereotypic behavior promote daily functioning in patients with schizophrenia. Aust N Z J Psychiatry, 2021:48674211038513. 10.1177/00048674211038513 34376088

[93] Bonfils KA (2019). Affective prosody and facial emotion recognition in first-episode schizophrenia: Associations with functioning & symptoms. Schizophr Res Cogn.

[94] Brunet-Gouet E (2021). Outcome prediction with a social cognitive battery: a multicenter longitudinal study. NPJ Schizophr.

[95] Corbera S (2021). Predictors of social functioning and quality of life in schizophrenia and autism spectrum disorder. Psychiatry Res.

[96] Engelstad KN (2017). Body language reading of emotion in schizophrenia: Associations with symptoms and functional outcome. Scand J Psychol.

[97] Frajo-Apor B (2021). The relationship between emotional intelligence and quality of life in schizophrenia and bipolar I disorder. Qual Life Res.

[98] Gürcan MB (2021). The Effects of Narrative and Movie Therapy on the Theory of Mind and Social Functioning of Patients with Schizophrenia. Noro Psikiyatr Ars.

[99] Hajdúk M (2018). Theory of mind - not emotion recognition - mediates the relationship between executive functions and social functioning in patients with schizophrenia. Psychiatr Danub.

[100] Hajdúk M (2020). Implicit and explicit processing of bodily emotions in schizophrenia. Cogn Neuropsychiatry.

[101] Kolavarambath R (2020). Emotion Recognition, Emotion Awareness, Metacognition, and Social Functioning in Persons with Schizophrenia. Indian J Psychol Med.

[102] Kurtz MM (2018). Cognition, social cognition and functional disability in early-stage schizophrenia: A study from southern India. Psychiatry Res.

[103] Le TP (2018). Neurocognitive and theory of mind deficits and poor social competence in schizophrenia: The moderating role of social disinterest attitudes. Psychiatry Res.

[104] Ludwig KA (2017). Social cognition psychometric evaluation (SCOPE) in people with early psychosis: A preliminary study. Schizophr Res.

[105] Park K, Choi KH (2018). Paranoia Symptoms Moderate the Impact of Emotional Context Processing on Community Functioning of Individuals with Schizophrenia. Community Ment Health J.

[106] Hariri AR (2002). The amygdala response to emotional stimuli: a comparison of faces and scenes. Neuroimage.

